# Identification and verification of three autophagy-related genes as potential biomarkers for the diagnosis of psoriasis

**DOI:** 10.1038/s41598-023-49764-0

**Published:** 2023-12-21

**Authors:** Ailing Zou, Yongjun Chen, Tangsheng Liu, Ting Yang, Bei Zhou

**Affiliations:** 1 Department of Dermatology, Huangshi Central Hospital, Affiliated Hospital of Hubei Polytechnic University, Huangshi, China; 2 Hubei Key Laboratory of Kidney Disease Pathogenesis and Intervention, Huangshi, Hubei China; 3 Department of Stomatology, Huangshi Central Hospital, Affiliated Hospital of Hubei Polytechnic University, Huangshi, China

**Keywords:** Diagnostic markers, Skin diseases

## Abstract

Psoriasis vulgaris is the most common form of the four clinical types. However, early diagnosis of psoriasis vulgaris is difficult due to the lack of effective biomarkers. The aim of this study was to screen potential biomarkers for the diagnosis of psoriasis. In our study, we downloaded the original data from GSE30999 and GSE41664, and the autophagy-related genes list from human autophagy database to identify differentially expressed autophagy-related genes (DERAGs) by R software. Then Gene Ontology (GO) and Kyoto Encyclopedia of Genes and Genomes (KEGG) pathway enrichment analysis were performed for DERAGs. DERAGs were validated by the other four databases (GSE13355, GSE14905, GSE6710, and GSE55201) to screen biomarkers with high diagnostic value for the early diagnosis of psoriasis vulgaris. Finally, DERAGs were verified in our clinical blood samples by ELISA. A total of 12 DERAGs were identified between 123 paired non-lesional and lesional skin samples from patients with psoriasis vulgaris. GO and KEGG enrichment analysis indicated the TORC2 complex was more enriched and the NOD-like receptor signaling pathway was mostly enriched. Three autophagy-related genes (*BIRC5*, *NAMPT* and *BCL2*) were identified through bioinformatics analysis and verified by ELISA in clinical blood samples. And these genes showed high diagnostic value for the early diagnosis of psoriasis vulgaris. We identified three autophagy-related genes (*BIRC5*, *NAMPT* and *BCL2*) with high diagnostic value for the early diagnosis of psoriasis vulgaris through bioinformatics analysis and clinical samples. Therefore, we proposed that *BIRC5*, *NAMPT* and *BCL2* may be as potential biomarkers for the early diagnosis of psoriasis vulgaris. In addition, *BIRC5*, *NAMPT* and *BCL2* may affect the development of psoriasis by regulating autophagy.

Psoriasis is a chronic inflammatory skin disease that affects 2–3% of the world’s population^1^. At present, effective treatment of the disease is limited, and it relapses easily, which usually leads to a significantly decreased quality of life, increased psychological stress, and obvious economic burden^2^. Of the four clinical types, psoriasis vulgaris is the most common form, with erythematous and scaly plaques as its main manifestations^3^, and accounts for nearly 90% of all psoriasis conditions^4^.

Signs and symptoms of psoriasis vulgaris are sometimes atypical and always require a differential diagnosis of eczema or pityriasis rosea. Several studies have been performed to elucidate the molecular mechanisms underlying psoriasis^5,6^. However, only a few studies have reported biomarkers for the diagnosis of psoriasis vulgaris. In this study, we attempted to identify potential biomarkers for the diagnosis of psoriasis vulgaris based on bioinformatics analysis.

One possible method is to search the GEO database for potential diagnostic biomarkers of psoriasis vulgaris, as large-scale free datasets in GEO currently make the analysis feasible. To date, many studies have focused on the potential mechanisms involved in psoriasis. For example, Zeng et al. reported that some transcription factors could play an important role in the pathogenesis of psoriasis^5^; Deng et al. found some key regulators of psoriasis^6^; and our team also identified 13 key apoptosis-related genes associated with psoriasis^7^.

Autophagy is a cellular process that involves the degradation and recycling of cellular components, such as damaged proteins, organelles, and other cytoplasmic contents^8^. Autophagy has been implicated in a number of diseases including psoriasis^9^. For example, LncRNA MEG3 enhance autophagy by PI3K/AKT/mTOR signalling pathway to inhibit psoriasis-like skin inflammation^10^.In addition, aurora kinase A promotes the psoriasis-related inflammation by regulating autophagy^11^. Therefore, understanding the mechanisms of autophagy and its regulation may have important implications for the occurrence and development of psoriasis. However, there are few studies focused on autophagy-related genes in the diagnosis of psoriasis. Exploring the potential autophagy-related genes of psoriasis will provide us potential biomarkers for the diagnosis of psoriasis.

Here, we analyzed large-scale public data to identify diagnostic biomarkers of psoriasis vulgaris using two GEO databases, one list of autophagy-related genes, and four validation GEO databases. First, we identified differentially expressed autophagy-related genes (DERAGs) between non-lesional (NL) and lesional (LS) skin samples from patients with psoriasis vulgaris. Then Gene Ontology (GO) and Kyoto Encyclopedia of Genes and Genomes (KEGG) pathway enrichment analysis were performed for DERAGs. We verified these genes using four GEO databases and finally acquired three autophagy-related genes, including *BIRC5*, *NAMPT*, and *BCL2*. In the end, our findings were validated in clinical blood samples using ELISA.

## Methods

### Microarray data and autophagy-related genes

Six datasets were downloaded from GEO (http://www.ncbi.nlm.nih.gov/geo/). Two GPL570 datasets, GSE30999^12^ and GSE41664^13^, which contained 123 paired non-lesional (NL) and lesional skin (LS) samples from patients with psoriasis vulgaris, were selected as test datasets. The other two GPL570 datasets, GSE13355^14^ and GSE14905^15^, which contained 85 normal skin (NN), 86 NL, and 91 LS samples; one GPL96 GSE6710 dataset^16^, which contained 13 paired NL and LS samples; and the GPL570 GSE55201 dataset^17^, which contained 30 blood samples from healthy controls and 44 blood samples from patients with psoriasis vulgaris, were selected as validation datasets (Table 1). A total of 222 autophagy-related genes (ARGs) were obtained from the Human Autophagy Database (http://www.autophagy.lu/index.html).

**Table 1:** Information for selected microarray datasets.

GEO accession	Platform	Samples			Source tissue
		NN	NL	LS	
GSE30999	GPL570		85	85	Skin
GSE41664	GPL570		38	38	Skin
GSE13355	GPL570	64	58	58	Skin
GSE14905	GPL570	21	28	33	Skin
GSE6710	GPL96		13	13(mild 5 moderate 7 severe 1)	Skin
GSE55201	GPL570	HC		PS	Blood
		30		44	

*NN* normal skin from control, *NL* non-lesional skin from psoriatic patient, *LS* lesional skin from psoriatic patient, *HC* healthy control, *PS* psoriasis patient.

### Identification of differentially expressed autophagy-related genes

The original data downloaded from GEO using the “GEOquery” package^18^ were pooled and normalized by the “sva” package. The clustering of data between two groups in GSE30999 and GSE41664 was verified by the “umap” package. The differentially expressed genes (DEGs) of GSE30999 and GSE41664 were identified using the “limma” package^19^. The volcano plot was visualized by the “ggplot2” package. The screening criteria were |log2FC|> 1 and padj < 0.05. Venn diagrams of DEGs and ARGs were used to identify differentially expressed autophagy-related genes (DERAGs). The heatmap of DERAGs was conducted by the “ComplexHeatmap” package^20^. Correlation analysis of DERAGs was performed using Spearman’s rank correlation coefficient.

### Gene ontology and Kyoto encyclopedia of genes and genomes enrichment analysis

Gene Ontology (GO) and Kyoto Encyclopedia of Genes and Genomes (KEGG) pathway enrichment analyses were conducted using the “GOplot” package^21^. The GO and KEGG results were presented as chord and loop diagrams, respectively^22^.

### Validation of differentially expressed autophagy-related genes in four datasets

The data of GSE13355 and GSE14905 were pooled, which contained 91 LS and 171 control samples. We first validated DERAGs in the two datasets with large samples. The GSE6710 dataset contained 13 paired NL and LS samples was selected as a validation dataset due to psoriasis severity grading, in which mild was five, moderate was seven, and severe was one (Table 1). The GSE55201 dataset was considered as a validation dataset because of blood samples, in which 7 treatment samples were omitted, 30 healthy controls and 44 psoriasis patients were retained.

### The data of psoriasis vulgaris patients and healthy controls

A total of 17 patients with psoriasis vulgaris and 15 age-matched healthy controls were recruited from the inpatient dermatology ward of Huangshi Central Hospital between March 2022 and May 2022. The severity of disease and PASI scores were also evaluated for patients. The details of patients and controls were showed in Table 2. The diagnosis of psoriasis vulgaris is based on classic clinical and pathological features. All patients met the diagnostic criteria for progressive psoriasis vulgaris. None of the patients took glucocorticoids, immunosuppressants, or retinoids within 3 months, and patients were excluded if they had other types of psoriasis, such as erythrodermic psoriasis. Blood samples were collected from 17 patients with psoriasis vulgaris and 15 healthy controls.

**Table 2:** Clinical features of cases and controls in the study.

Vabriables	Psoriasis vulgaris (n = 17)	Control (n = 15)	*P* value
Age (years)	43.47 ± 14.14	40.27 ± 11.56	0.49
Sex (male/female)	9/8	8/7	
Severity grading			
Mild	7		
Moderate-severe	10		
PASI	12.29 ± 6.16	0	< 0.0001

Data are presented as mean ± SD.

### Enzyme-linked immunosorbent assay

The levels of *BIRC5*, *NAMPT*, and *BCL2* in the blood samples from each participant were measured using human survivin (BIRC5) ELISA kit (ELK Biotechnology, Wuhan, China), human visfatin (NAMPT) Enzyme-linked immunosorbent assay ELISA kit (ELK Biotechnology, Wuhan, China), and human BCL2 ELISA kit (ELK Biotechnology, Wuhan, China) according to the manufacturer’s instructions.

### Statistics analysis

Data analysis and visualization were conducted using R software (3.6.3). Correlation analysis was performed using Spearman’s correlation coefficient. When the samples satisfied the normality test, an Independent-Samples T-test was used for the two groups. When the samples did not satisfy the normality test, the Mann–Whitney U test was used for the two groups. When the samples satisfied the normality test, one-way ANOVA was used for multiple groups. If the samples did not satisfy the normality test, the Kruskal–Wallis test was used for multiple groups. The receiver operating characteristic (ROC) curve was also performed using R software (*P < 0.05; **P < 0.01; ***P < 0.001).

### Ethics approval and consent to participate

This study was approved by the Medical Ethics Committee of Huangshi Central Hospital, Hubei, China (1.0.2022.03.31), and informed consent was obtained from all the participants. The experimental scheme was approved by the academic committee of Huangshi Central Hospital, and the experimental methods were carried out in accordance with the guidelines of the academic committee.

## Results

### Data collation and differentially expressed autophagy-related genes screening

**Figure 1:**
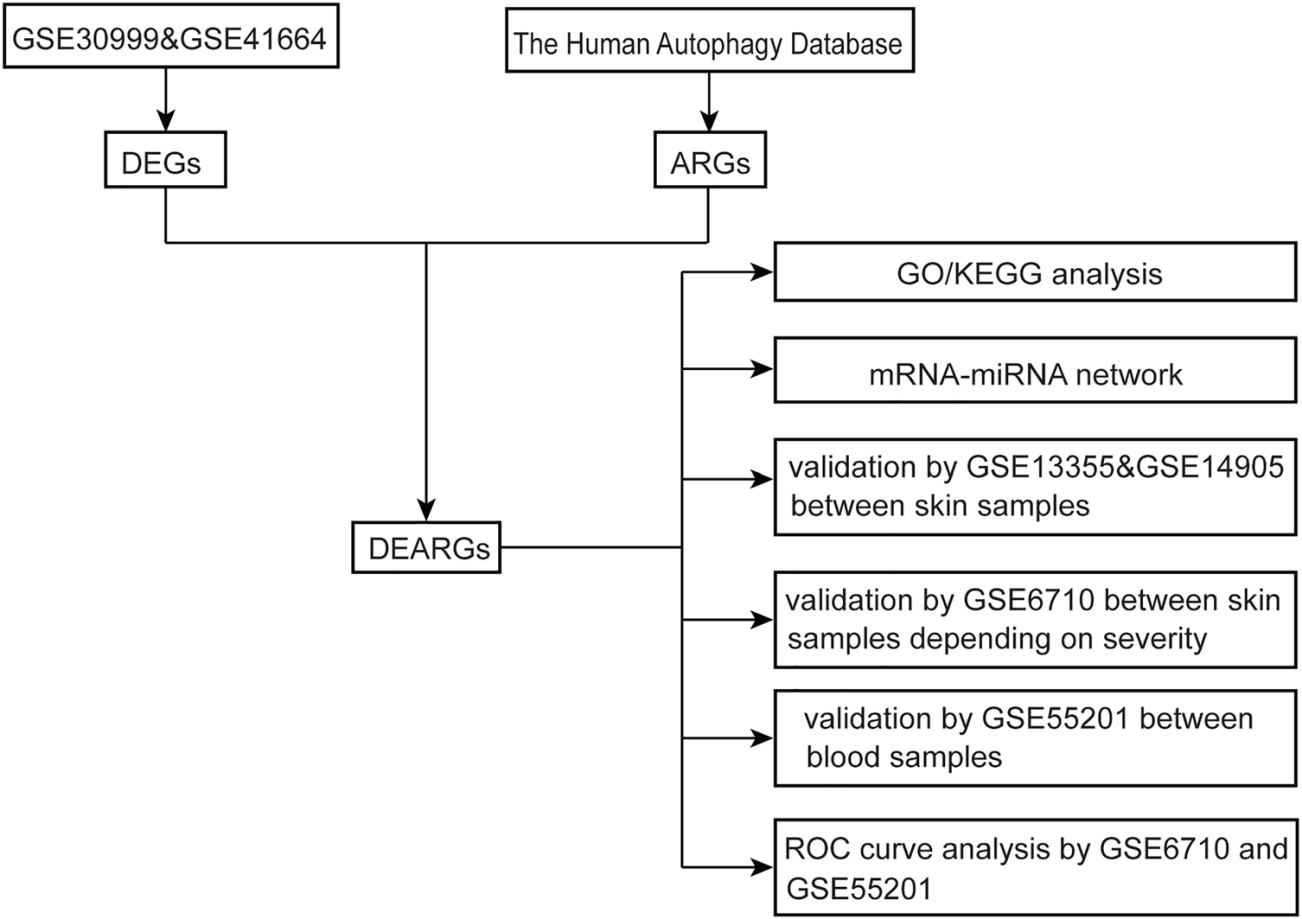
The flowchart of the study. DEGs, differentially expressed genes; ARGs, autophagy-related genes; DERAGs, differentially expressed autophagy-related genes; GO, Gene Ontology; KEGG, Kyoto Encyclopedia of Genes and Genomes.

**Figure 2:**
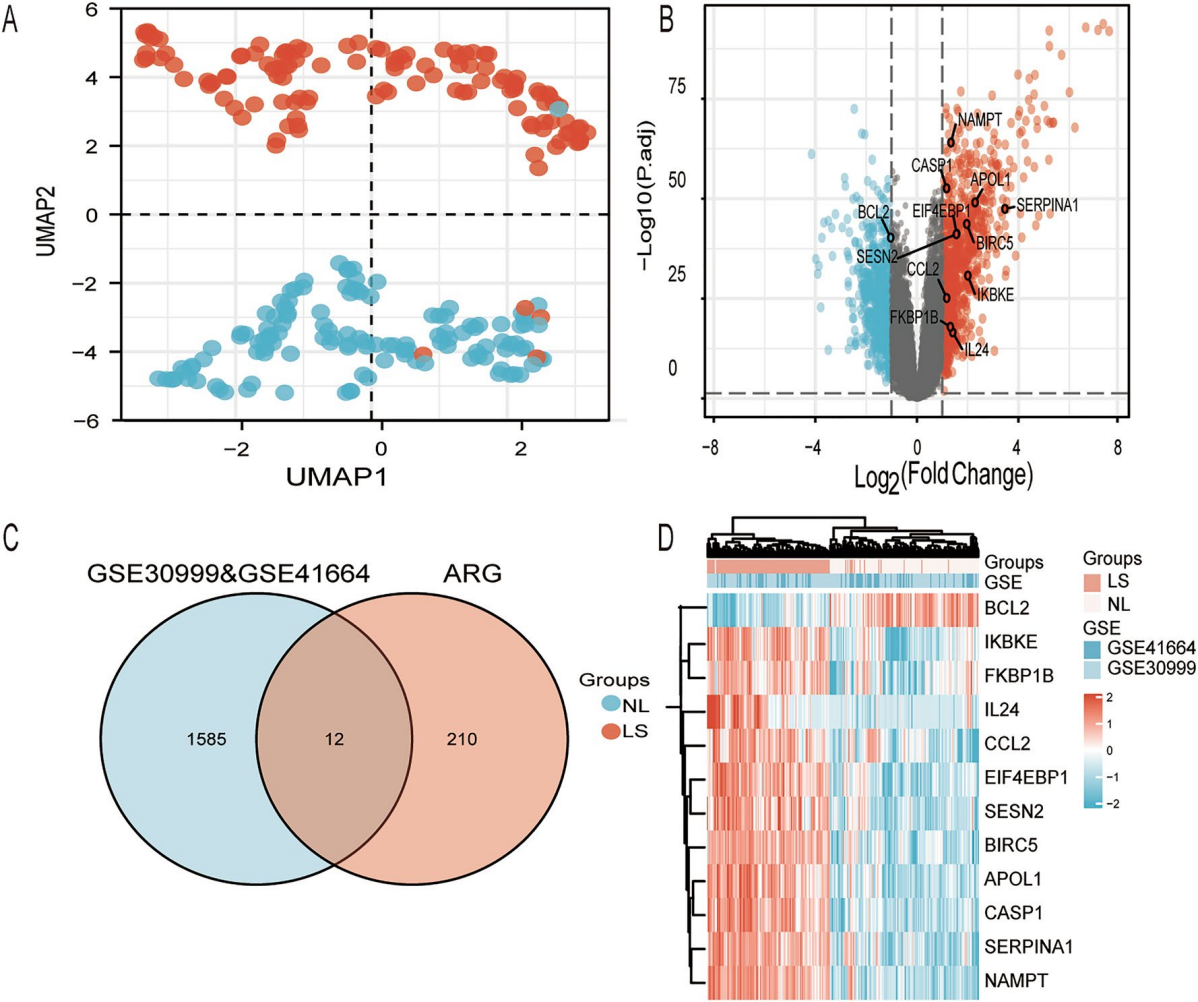
Screening of differentially expressed autophagy-related genes (DERAGs) in GSE30999 and GSE41664. (**A**) Uniform manifold approximation and projection (UMAP) for GSE30999 and GSE41664. (**B**) Volcano plot of 1597 DEGs including 12 DERAGs. The red and blue dots represent the up-regulated and down-regulated genes, respectively. (**C**) Venn diagram of the 12 DERAGs. (**D**) Heatmap of the 12 DERAGs in lesional skin (LS) and non-lesional (NL) samples.

**Figure 3:**
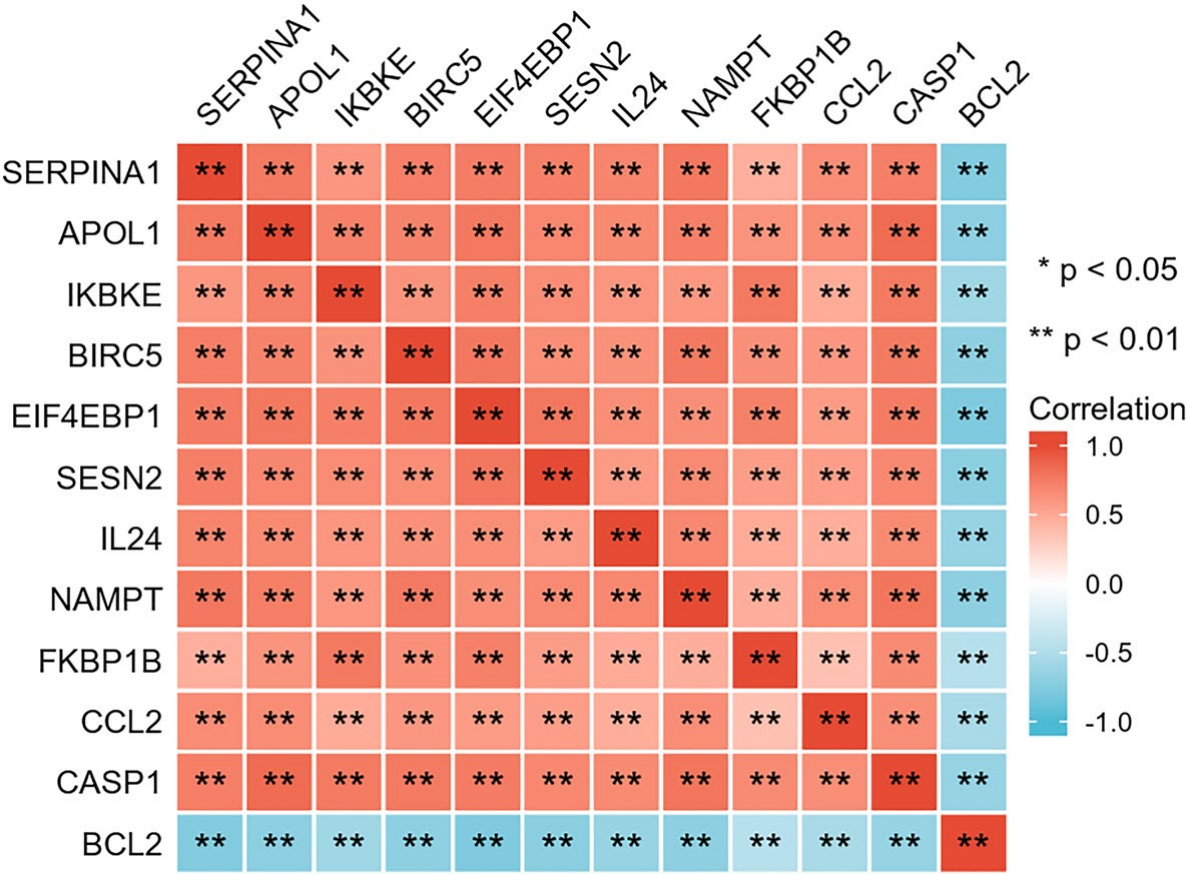
Spearman correlation analysis of the 12 DERAGs. ^*^P < 0.05; ^**^P < 0.01.

The flowchart of the study is shown in Fig. 1, and the collated datasets downloaded from the GEO are shown in Table 1. Uniform manifold approximation and projection (UMAP) was used for dimensionality reduction and cluster identification of the GSE30999 and GSE41664 datasets (Fig. 2A). The datasets GSE30999 and GSE41664 were selected to identify the differentially expressed genes, and a total of 1597 DEGs were identified. A volcano plot was used to visualize the DEGs, as shown in Fig. 2B. Venn diagrams were used to identify DERAGs, and 12 DERAGs were acquired, including 11 up-regulated genes (*SERPINA1*, *APOL1*, *IKBKE*, *BIRC5*, *SESN2*, *EIF4EBP1*, *FKBP1B*, *NAMPT*, *IL24*, *CASP1*, *CCL2*) and 1 down-regulated gene (*BCL2*) (Fig. 2C, Table 3). A complex heatmap of the 12 DERAGs is shown in Fig. 2D. The correlation analysis of the 12 DERAGs showed strong correlations between them (Fig. 3).

**Table 3:** The 12 differentially expressed autophagy-related genes in LS compared to NL.

Gene symbol	logFC	Changes	*P* value	Adj. *P* value
SERPINA1	4.226705882	Up	4.61E−50	3.49E−48
APOL1	2.683176471	Up	9.64E−52	8.46E−50
IKBKE	2.486235294	Up	1.04E−32	1.79E−31
BIRC5	2.146352941	Up	4.17E−46	2.14E−44
SESN2	1.927294118	Up	1.98E−43	7.85E−42
EIF4EBP1	1.880117647	Up	1.48E−43	5.96E−42
FKBP1B	1.760235294	Up	1.96E−19	1.21E−18
NAMPT	1.432	Up	2.82E−67	8.84E−65
IL24	1.416235294	Up	5.08E−18	2.84E−17
CASP1	1.338	Up	2.13E−55	2.47E−53
CCL2	1.305294118	Up	6.82E−27	7.25E−26
BCL2	− 1.168705882	Down	1.52E−42	5.65E−41

*LS* lesional skin from psoriatic patient, *NL* non-lesional skin from psoriatic patient.

### Gene ontology and Kyoto encyclopedia of genes and genomes enrichment analysis

**Figure 4:**
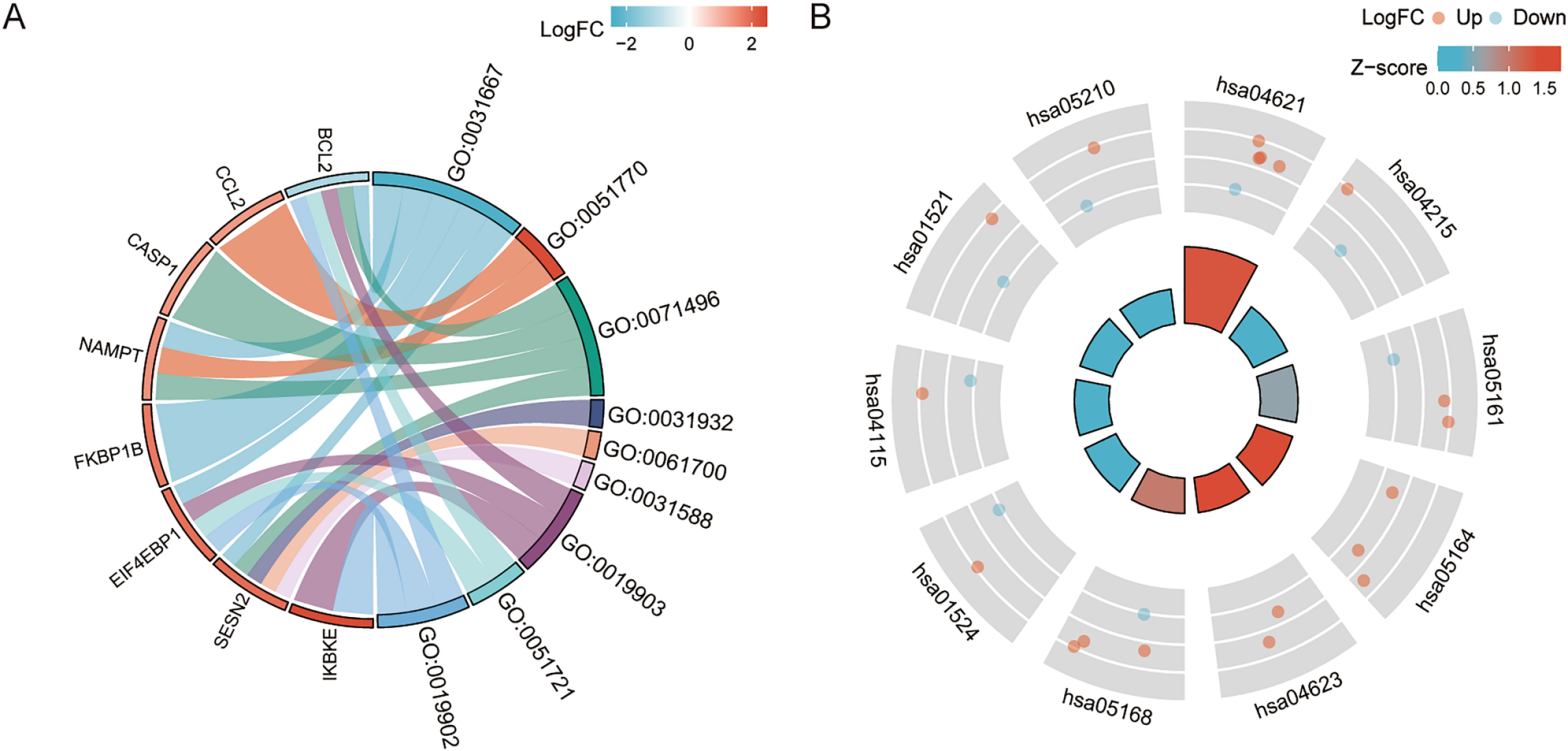
GO and KEGG enrichment analyses of DEGs. (**A**) The chord plot showing the top 9 GO terms. (**B**) The loop diagram showing the 10 top pathways.

The GO and KEGG pathways were analyzed for the datasets GSE30999 and GSE41664 using the R software GOplot package. The GO results showed the top three terms for cellular component (CC), biological process (BP), and molecular function (MF) (Table S1). The top nine GO terms were selected based on a *p* value < 0.05 and were drawn in a chord plot (Fig. 4A). The KEGG pathway results indicated that the most enriched pathway was the NOD-like receptor signaling pathway (Table S1). The top 10 pathways were also selected based on a *p* value < 0.05 and were drawn in a loop diagram (Fig. 4B).

### Validation of 12 DERAGs in four datasets

**Figure 5:**
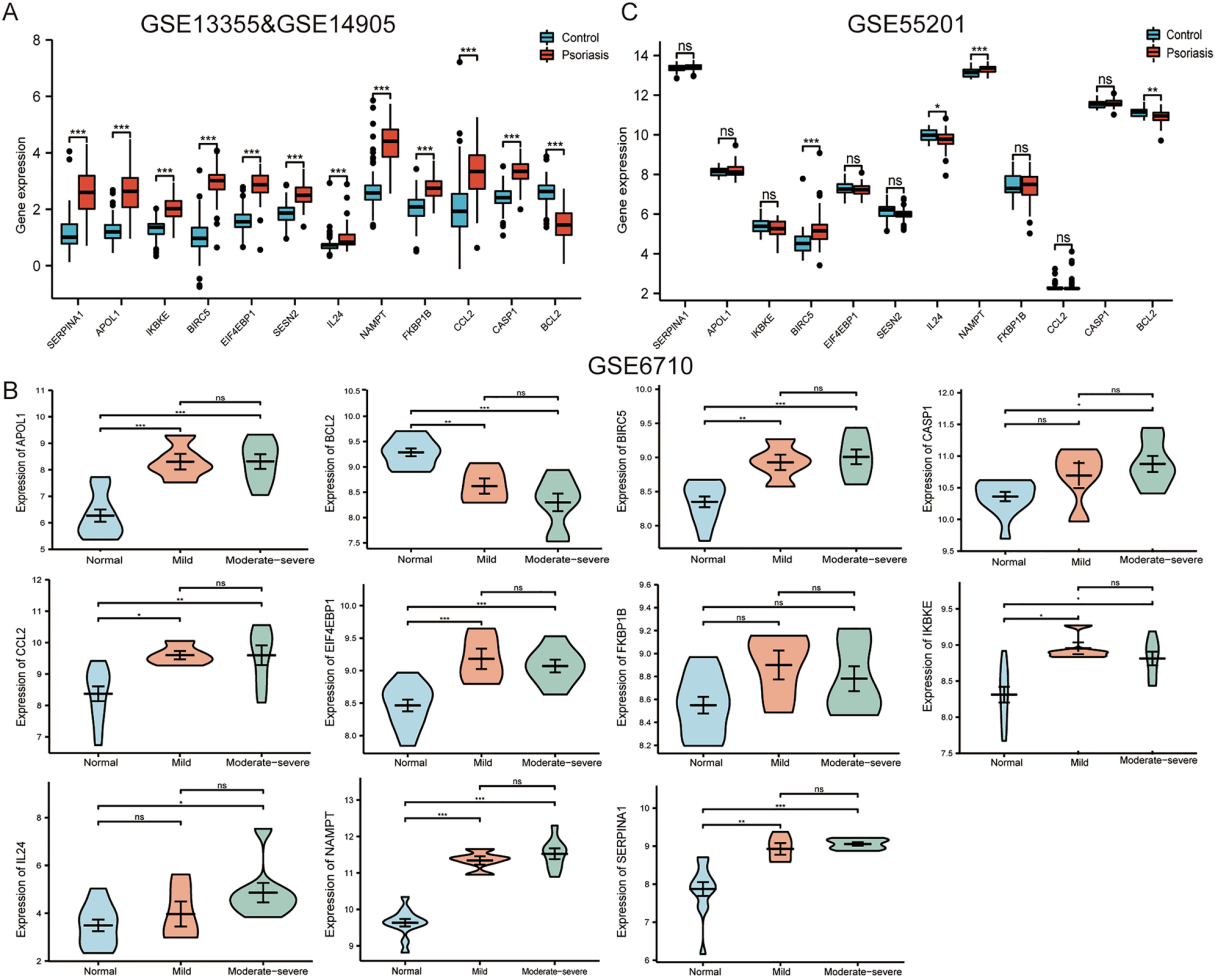
Verification of the 12 DERAGs in four datasets. (**A**) Verification by GSE13355 and GSE14905. Compared with LS samples and NN and NL skin samples, 11 DERAGs were significantly increased and *BCL2* was significantly decreased. (**B**) Verification by GSE6710. Compared with LS and NL samples, 7 DERAGs (*APOL1*, *BIRC5*, *CCL2*, *EIF4EBP1*, *IKBKE*, *NAMPT* and *SERPINA1*) were significantly increased and *BCL2* were significantly decreased. There were no statistical significances in their expression levels between mild and moderate-severe psoriasis samples. (**C**) Verification by GSE55201. Compared with psoriatic and healthy blood samples, *BIRC5* and *NAMPT* were significantly increased and *BCL2* and *IL24* were significantly decreased. ^*^P < 0.05; ^**^P < 0.01; ^***^P < 0.001; ns, no significant difference.

The data were analyzed using R software (3.6.3). The pooled results of GSE13355 and GSE14905 datasets showed that the expression levels of 11 DERAGs were increased significantly and the expression level of *BCL2* was decreased significantly when compared with LS samples and NN and NL samples, which were consistent with the results of two test datasets (Fig. 5A). Similarly, the results of the GSE6710 dataset demonstrated that the expression levels of 7 DERAGs (*APOL1*, *BIRC5*, *CCL2*, *EIF4EBP1*, *IKBKE*, *NAMPT*, and *SERPINA1*) were increased significantly and the expression level of *BCL2* was decreased significantly when compared with LS samples and NL samples (Fig. 5B). In addition, we found that there were no statistical significances in their expression levels between mild and moderate-severe psoriasis samples (Fig. 5B). Furthermore, there was no expression of *SESN2* and were no statistical significances of 3 DERAGs (*CASP1*, *FKBP1B*, and *IL24*) in the GSE6710 dataset. Nevertheless, the results of the GSE55201 dataset indicated that the expression levels of *BIRC5* and *NAMPT* were significantly higher and the expression levels of *BCL2* and *IL24* were significantly lower in psoriatic blood samples than healthy blood samples (Fig. 5C). In summary, we validated 3 DERAGs in skin and blood samples by four different datasets. Specifically, *BIRC5* and *NAMPT* were up-regulated, and *BCL2* expression was down-regulated.

### ROC curves of 3 DERAGs in GSE6710 and GSE55201 datasets

**Figure 6:**
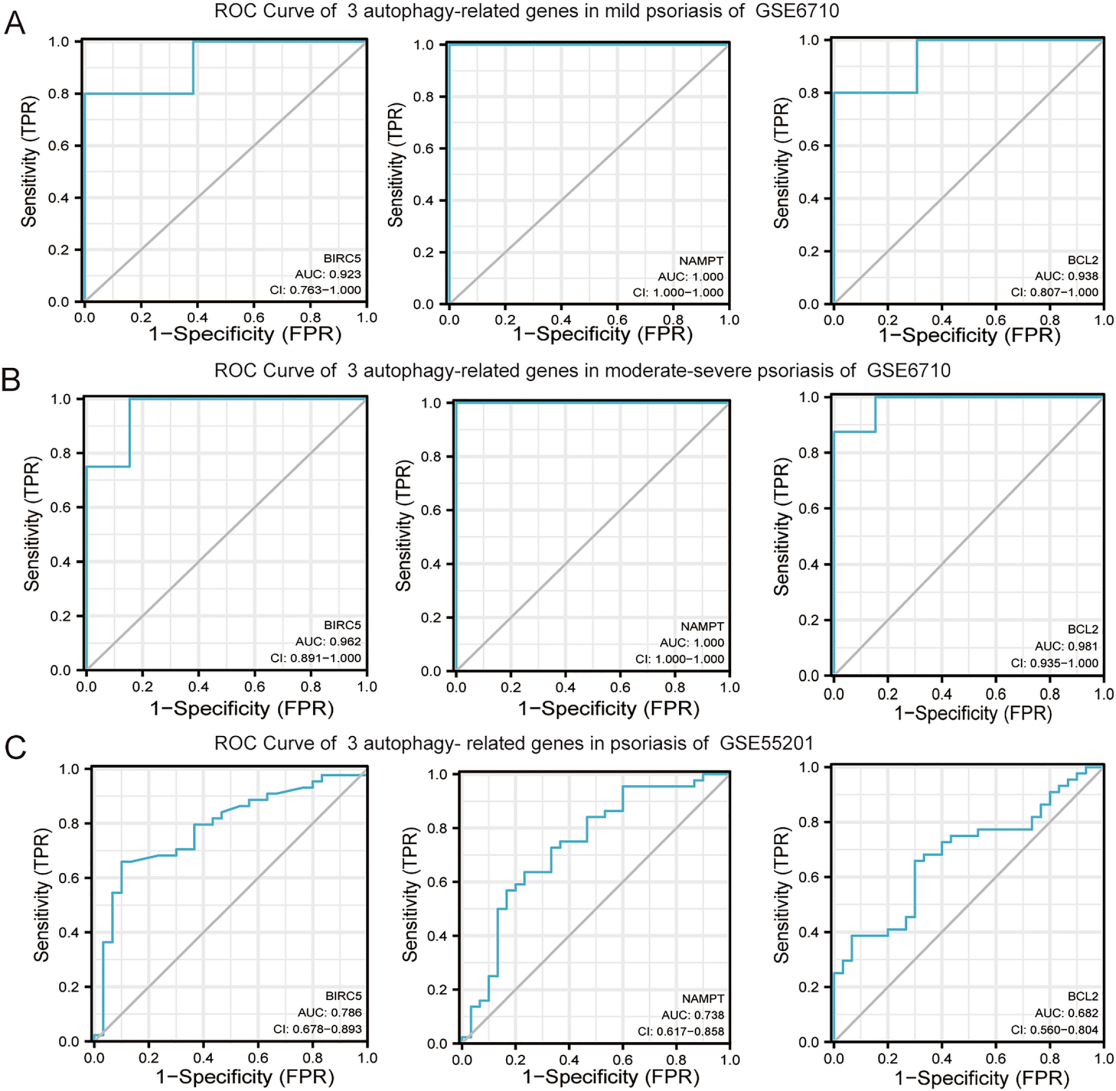
ROC Curves of the 3 DERAGs in two datasets. (**A**) ROC Curves of *BIRC5*, *NAMPT*, and *BCL2* in mild psoriasis skin samples. (**B**) ROC Curves of *BIRC5*, *NAMPT*, and *BCL2* in moderate-severe psoriasis skin samples. (**C**) ROC Curves of *BIRC5*, *NAMPT*, and *BCL2* in blood samples.

We used R software to draw ROC curves for *BIRC5*, *NAMPT*, and *BCL2*). The area under the curve (AUC) is an indicator of the diagnostic effect; the greater the value, the better the diagnostic effect. In the GSE6710 dataset, *BIRC5*, *NAMPT*, and *BCL2* had high diagnostic values in both mild and moderate-severe psoriasis vulgaris samples (Fig. 6A, B). All three genes had AUC values greater than 0.9, and *NAMPT* had the highest diagnostic value (AUC:1.000) in psoriatic skin samples (Fig. 6A, B). In the GSE55201 dataset, *BIRC5*, *NAMPT*, and *BCL2* had high diagnostic values in psoriatic blood samples and *BIRC5* had the highest diagnostic value (AUC:0.786) (Fig. 6C).

### Verification of 3 DERAGs in our clinical blood samples

**Figure 7:**
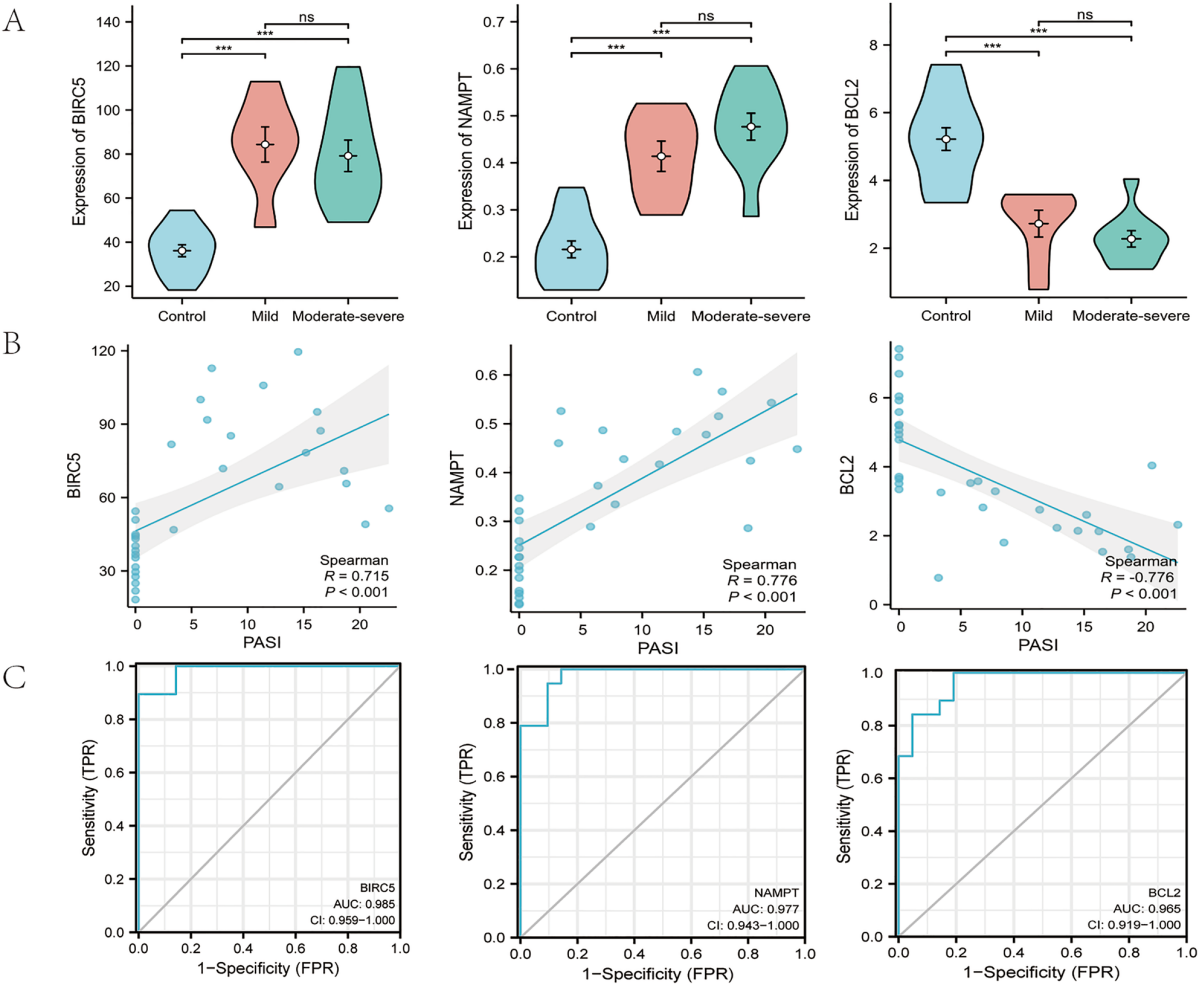
Verification of the 3 DERAGs in our clinical blood samples. (**A**) Compared with psoriatic and healthy blood samples, *BIRC5* and *NAMPT* was significantly increased, *BCL2* was significantly decreased (*P* < 0.001). No statistical significances of their expression levels existed in blood samples of mild and moderate-severe psoriasis. (**B**) The expression levels of *BIRC5* and *NAMPT* were positively correlated with PASI scores, and the expression level of *BCL2* was negatively correlated with PASI scores. (**C**) ROC Curves of *BIRC5* (AUC:0.985), *NAMPT* (AUC:0.977) and *BCL2* (AUC:0.965) in clinical blood samples. ^*^P < 0.05; ^**^P < 0.01; ^***^P < 0.001.

The expression levels of *BIRC5*, *NAMPT*, and *BCL2* were tested by ELISA in the blood samples of patients with psoriasis vulgaris and healthy controls. The expression levels of *BIRC5* and *NAMPT* were significantly higher, and the expression level of *BCL2* was significantly lower in psoriatic blood samples than healthy blood samples (Fig. 7A). At the same time, no statistical significances of their expression levels existed in in blood samples of mild and moderate-severe psoriasis (Fig. 7A). As shown in Fig. 7B, the expression levels of *BIRC5* and *NAMPT* were positively correlated with PASI scores, and the expression level of *BCL2* was negatively correlated with PASI scores. ROC curves illustrated that *BIRC5*, *NAMPT*, and *BCL2* had high diagnostic values in psoriatic blood samples and *BIRC5* had the highest diagnostic value (AUC:0.985) (Fig. 7C).

## Discussion

The different types of psoriasis include psoriasis vulgaris, pustular psoriasis, erythrodermic psoriasis, and arthritic psoriasis^23^. Psoriasis vulgaris is the most common type, manifesting as an erythematous scaly plaque^3^. However, the clinical presentation of psoriasis vulgaris is sometimes atypical, resulting in frequent misdiagnosis^24^. Although the gold standard of diagnosis is skin biopsy, it may not be accepted by patients due to its invasiveness and the long waiting period for pathologic result. Thus, it is imperative to identify effective biomarkers for the early diagnosis of psoriasis vulgaris.

In our study, we identified 1597 DEGs, including 834 upregulated and 763 downregulated genes, by comparing gene expression in 123 paired non-lesional and lesional psoriatic skin samples. GO enrichment and KEGG pathway analyses were performed. The GO enrichment analysis was more enriched in the TORC2 complex. It is worth noting that mTORC2 is an inhibitor of autophagy^25^. The KEGG pathway was mostly enriched in the NOD-like receptor signaling pathway. Yang et al. also revealed that KEGG pathway analysis was mainly enriched in the NOD-like receptor axis using the GEO datasets of psoriasis^26^.

We identified 12 DERAGs using Venn diagrams. After the 12 DERAGs were validated using four GEO datasets, we acquired three DERAGs (*BIRC5*, *NAMPT*, and *BCL2*). In the four datasets, compared with their expression levels in psoriatic and control skin samples, *BIRC5* and *NAMPT* were significantly upregulated, and *BCL2* was significantly downregulated; compared with their expression levels in psoriatic and control blood samples, *BIRC5* and *NAMPT* were also significantly upregulated, and *BCL2* was significantly downregulated. Further, ROC curve analysis showed that the three genes have good diagnostic values for both psoriasis vulgaris skin and blood samples. To increase the reliability of the above results, we verified the expression levels of *BIRC5*, *NAMPT* and *BCL2* in our clinical blood samples. These results were consistent with our expectations that *BIRC5* and *NAMPT* were upregulated and *BCL2* was downregulated in psoriatic blood samples. Additionally, there was no significant differences in their expression levels between mild and moderate-severe psoriasis patients. The three genes had good diagnostic values for clinical blood samples. Therefore, we hypothesized that *BIRC5*, *NAMPT*, and *BCL2* might serve as biomarkers for the early diagnosis of psoriasis vulgaris.

There are three commonly established pathways of apoptosis: extrinsic, intrinsic, and granzyme/perforin pathways^27^. The B cell lymphoma-2 (BCL2) family members are important components of the intrinsic pathway^28^. It is known to all that *BCL2* is a key member of the BCL2 family, which can inhibit apoptosis and promote cell survival, and it usually has abnormal expression or function in almost all tumors^29^. Many studies have reported contradictory results regarding *BCL2* expression in psoriatic skin. Kastelan et al. reported the upregulated expression of *BCL2* in psoriatic skin^30^. However, Batinac et al. and Gündüz et al. showed downregulated expression of *BCL2* in psoriatic skin compared with normal skin^31,32^. The *BCL2* expression in our blood samples was consistent with that in the latter.

Baculoviral IAP repeat-containing 5 (BIRC5), also known as survivin, API4, or EPR-1, is an inhibitor of apoptosis^33^. The upregulated expression of *BIRC5* can be observed in many types of cancers, such as breast cancer and esophageal cancer^34,35^. *BIRC5* may play an important role in psoriasis pathogenesis because of its effects on apoptosis^36^. Several studies have indicated significantly upregulated expression of *BIRC5* in psoriatic skin and blood samples compared to controls^36,37^. The *BIRC5* expression in our blood samples was consistent with theirs.

Nicotinamide phosphoribosyltransferase (NAMPT), also known as PBEF and visfatin, is considered an enzyme that is involved in nicotinamide adenine dinucleotide (NAD) biosynthesis^38^. NAMPT exists in two major forms: an intracellular form (iNAMPT) and an extracellular form (eNAMPT)^38^. iNAMPT plays a key role in intracellular NAD levels. eNAMPT also acts as an immunomodulatory cytokine in multiple pathways in addition to its enzymatic activity^39^. It has been reported that eNAMPT can be involved in various metabolic disorders and cancer^40,41^. Several studies have revealed that the NAMPT-mediated NAD salvage pathway may contribute to the pathogenesis of psoriasis^42,43^. Mercurio et al. reported that *NAMPT* expression was upregulated in psoriatic skin compared with normal skin^42^. The *NAMPT* expression in our blood samples was consistent with their results.

Therefore, we considered three genes (*BIRC5*, *NAMPT*, and *BCL2*) as potential biomarkers for the early diagnosis of psoriasis vulgaris. However, there were some limitations in this study. First, several datasets may have resulted in unavoidable batch differences during the analysis. Second, since different datasets have different analytical methods, quite big differences maybe exist in the list of significantly changed genes. Third, the sample size of the study is rather small (17 cases and 15 controls), which weakens the strength of the results. Fourth, we also verified the three autophagy-related genes using RT-qPCR in clinical psoriasis vulgaris samples. But the mRNA expression levels of these genes in psoriatic skin samples were not statistically significant compared with those in the control groups, possibly because of insufficient psoriatic skin samples and unqualified storage conditions (n = 10). Thus, we plan to increase the sample size and improve the preservation standards in the next step for further study.

## Conclusion

In our study, we first identified 12 differentially expressed autophagy-related genes in psoriasis vulgaris using bioinformatics analysis. We then acquired three autophagy-related genes (*BIRC5*, *NAMPT*, and *BCL2*). Finally, the three autophagy-related genes were successfully validated in clinical blood samples. Therefore, we hypothesized *BIRC5*, *NAMPT*, and *BCL2* as potential biomarkers for the early diagnosis of psoriasis vulgaris using bioinformatics analysis and clinical samples. Moreover, the three autophagy-related genes may influence the pathogenesis of psoriasis by regulating autophagy. These results may provide clues for the development of new targeted therapies.

### Supplementary Information

Supplementary Information.

## Data Availability

The gene expression profiles of GSE30999, GSE41664, GSE13355, GSE14905, GSE6710 and GSE55201 were downloaded from Gene Expression Omnibus GEO) (https://www.ncbi.nlm.nih.gov/geo/query/acc.cgi?acc=GSE30999, https://www.ncbi.nlm.nih.gov/geo/query/acc.cgi?acc=GSE41664, https://www.ncbi.nlm.nih.gov/geo/query/acc.cgi?acc=GSE13355, https://www.ncbi.nlm.nih.gov/geo/query/acc.cgi?acc=GSE14905, https://www.ncbi.nlm.nih.gov/geo/query/acc.cgi?acc=GSE6710, and https://www.ncbi.nlm.nih.gov/geo/query/acc.cgi?acc=GSE55201).
