# Supplementary material for: Identification and verification of three autophagy-related genes as potential biomarkers for the diagnosis of psoriasis
Source: Sci Rep. 2023 Dec 21;13:22918. doi: 10.1038/s41598-023-49764-0 (PMC10739819; doi:10.1038/s41598-023-49764-0)
Supplement: Supplementary file 1 — Supplementary Information. [file 41598_2023_49764_MOESM1_ESM.docx]

| **ONTOLOGY** | **ID** | **Description** | ***P*-value** |
| --- | --- | --- | --- |
| BP | GO:0031667 | response to nutrient levels | 9.06427E-06 |
| BP | GO:0051770 | positive regulation of nitric-oxide synthase biosynthetic process | 3.95805E-05 |
| BP | GO:0071496 | cellular response to external stimulus | 4.71095E-05 |
| CC | GO:0031932 | TORC2 complex | 0.006676083 |
| CC | GO:0061700 | GATOR2 complex | 0.007280969 |
| CC | GO:0031588 | nucleotide-activated protein kinase complex | 0.008489729 |
| MF | GO:0019903 | protein phosphatase binding | 0.000101178 |
| MF | GO:0051721 | protein phosphatase 2A binding | 0.000206715 |
| MF | GO:0019902 | phosphatase binding | 0.000230688 |
| KEGG | hsa04621 | NOD-like receptor signaling pathway | 2.21646E-06 |
| KEGG | hsa04215 | Apoptosis - multiple species | 0.000818182 |
| KEGG | hsa05161 | Hepatitis B | 0.001161392 |
| KEGG | hsa05164 | Influenza A | 0.001358065 |
| KEGG | hsa04623 | Cytosolic DNA-sensing pathway | 0.003148201 |
| KEGG | hsa05168 | Herpes simplex virus 1 infection | 0.003327329 |
| KEGG | hsa01524 | Platinum drug resistance | 0.004204915 |
| KEGG | hsa04115 | p53 signaling pathway | 0.004204915 |
| KEGG | hsa01521 | EGFR tyrosine kinase inhibitor resistance | 0.004907797 |
| KEGG | hsa05210 | Colorectal cancer | 0.005791921 |

**Table S1.** GO and KEGG enrichment summary
